# 

**Published:** 1870-07

**Authors:** 


*CONTENTS:*

ORIGINAL COMMUNICATIONS:

ART. I. A Uterine Polypus of eighteen years' growth successfully removed. By J. W. Brooks, M. D. .......... 385
" II. Clinical Experiences in Private Practice, from a physical basis. By Z. C. McElroy, M. D. ..... 388
" III. Treatment of Conjunctivitis Neonatorum. By E. L. Holmes. 394
" IV. Miscellaneous Practice. By F. B. Norcom, A. M., M. D. ...... 397
" V. Clinical Lectures. By Jno. E. Owens, M. D. .......... 408
" VI. Nasal Catarrh. By M. F. Potter, M. D. .......... 416

ORIGINAL TRANSLATIONS:

ART. I. Legros and Onimus upon the Influence of Electric Currents upon the Nervous System. Continued... 419

REVIEW:

Barnes' Obstetric Operations.......... 428

EDITOR'S BOOK TABLE.......... 435

Basham's Renal Diseases. Gray's Anatomy. Pamphlets.

EDITORIAL: 437

Fees for Life Insurance Examinations. Medical Experts. Magnetic Springs.

LOOT: 439

Intestinal Puncture in Tympanitis. M. Auzias Turenne on Rabies. New Remedies for Burns. Chloral and Chloroform. Management of Pertussis. Cancrum Oris. Almost Complete Ossification of the Human Body. Rip Van Winkle, M. D.

